# Transformation of the Taiwan Biobank 3.0: vertical and horizontal integration

**DOI:** 10.1186/s12967-020-02451-4

**Published:** 2020-08-06

**Authors:** Jui-Chu Lin, Wesley Wei-Wen Hsiao, Chien-Te Fan

**Affiliations:** 1 College of Liberal Arts and Social Sciences, National Taiwan University of Science and Technology, Taipei, Taiwan, ROC; 2 Graduate Institute of Applied Science and Technology, National Taiwan University of Science and Technology, Taipei, Taiwan, ROC; 3 Law & Technology Innovation Center, National Taiwan University of Science and Technology, Taipei, Taiwan, ROC; 4 Ethical, Legal and Social Implications (ELSI) Working Task of the Taiwan Biobank, Taipei, Taiwan, ROC; 5 Department of Chemical Engineering, National Taiwan University of Science and Technology, Taipei, Taiwan, ROC; 6 Institute of Law for Science and Technology, National Tsing Hua University, Hsin-Chu, Taiwan, ROC

**Keywords:** Biobank 3.0, Vertical integration, Horizontal integration, Artificial intelligence (AI), Big Data, Ethical, Legal and Social Implications (ELSI)

## Abstract

Researchers expect a high quality of biospecimens/data and value-added services from biobanks. Therefore, the concept of “biobank 3.0” was introduced so that biobanks could better meet the needs of stakeholders and maintain sustainable operations. Theoretically, the Taiwan Biobank (TWB) has already gone through the concepts of biobank 1.0 and 2.0. However, three challenges still need to be addressed before it can be transformed into a new generation of the TWB (namely, the TWB 3.0): (1) the difficulty of integrating other biobanks’ resources, (2) the efficiency and effectiveness of the release and use of biospecimens/data, and (3) the development of income and revenue models of sustainability. To address these issues, this paper proposes a framework for the TWB 3.0 transformation based on a dual-pillar approach composed of a “physically” vertical integration driven by the TWB and a “virtually” horizontal network led by the National Health Research Institutes (NHRI) of Taiwan. Using prominent biobanks such as the Biobanking and BioMolecular Resources Research Infrastructure-European Research Infrastructure Consortium (BBMRI-ERIC), the UK Biobank, and the National Institutes of Health (NIH)’s All of Us Research Program as models, the TWB can strengthen its on-going TWB 2.0 operations in regional and/or international collaboration, increase the value of data collected and develop closer relationships with biobank participants and users. To these ends, the authors highlight key issues that include, but are not limited to, the harmonization of relevant ELSI standards for various biobanks’ integrations; the value-added services and the efficiency of Big Data Era related research and/or precision medicine development, and financial concerns related to biobank sustainability. This paper concludes by discussing how greater participant engagement and the uptake of Information Technology (IT) and Artificial Intelligence (AI) applications can be used in partnership with vertical and horizontal integration as part of a four-pronged approach to promote biobank sustainability, and facilitate the TWB 3.0 transformation.

## Introduction

Biobanks are considered to be one of the most important infrastructures for translating biomedical research and health data into practice and developing a better understanding of “precision medicine” [1]. Based on statistical analysis, the global biobanking market was valued at USD$ 1.54 billion in 2016, and is projected to reach USD$ 2.88 billion by 2025 at a compound annual growth rate (CAGR) of 7.2%. Furthermore, the global market for biobanking technologies is expected to increase from its valuation of $198.2 billion in 2016 to $240.2 billion in 2021, with a 5-year CAGR of 3.9% (the private sector is projected to increase from it’s 2016 valuation of $78.5 billion to $93.7 billion in 2021, with a 5-year CAGR of 3.6%). In addition, population biobanking is expected to reach $76.7 billion in 2021, up from $57.8 billion in 2016, with a 5-year CAGR of 5.8% [2]. Innovations in precision medicine, the increased incidence of chronic diseases, and advances in drug discovery and development are just a few of the reasons why, in recent years, biobanks have become indispensable. However, in order to ensure their long-term development, investment is required. In response to market demands, many countries have established a number of biobanks over the past 10 years. However, some of these initiatives have failed or shut down because of insufficient funds [3]. Thus, one of the most pressing issues facing biobanks in recent years has been related to how to ensure a stable source of funding. Many biobanks have not yet found a strategy to address sustainability issues. This has posed a challenge to the overall viability and usefulness of biobanks, which require substantial resources and sound business models in order to keep them running and evolving in health care [4, 5].

Hence, biobank sustainability has become a pressing issue [6]. It is evident that biobanks need to be more focussed on developing and maintaining sustainable business practices. However, the goal of “sustainable management” remains a critical challenge for biobanks around the world. The Taiwan biobank (TWB), which was established in 2012 and has been officially open for public access since 2014, has become the benchmark for all human biological databases in Taiwan [1]. So far, the TWB has collected the specimens and biological information of more than 130,000 participants and tracked them on a regular basis (every 2–4 years) so as to analyze the causes and/or mechanisms of chronic diseases. However, as a national-level biobank, the TWB needs to be able to operate in a sustainable fashion in order to maximize its usability and meet societal expectations. Therefore, the TWB requires a forward-looking approach that makes its human biological database attractive and useful to all sectors, including industry, government, academia, and research. Consequently, it is time to consider transforming biobanking practices through the formation of a novel approach.

Recently, the Taiwanese government has expressed concern that the country’s biobanks are being under-utilized. As a result, the usability of the TWB and 32 other biobanks in Taiwan has attracted a considerable amount of attention. Accordingly, the government believes that customizing value-added services to increase competitiveness is an important aspect of the management and application of biobanks. In addition to the TWB, the National Health Research Institutes (NHRI) offers a “virtually” horizontal integration platform promotion, which works in conjunction with TWB’s “physically” vertical integration that connects recruited data and clinical trial usages. When taken together, this dual approach facilitates the TWB 3.0 transformation. However, three outstanding issues remain. First, how will all available resources of the 33 biobanks in Taiwan be “efficiently” and “effectively” integrated for sustainable development when there is a lack of harmonization related to biobanking standards and regulations? Second, how can the efficiency of the whole biobanking process be improved (e.g., recruitment, acquisition, release and access)? Third, is it possible to develop a win–win scenario for an integrated platform and partner biobanks? Through the investigation of a variety of internationally renowned biobanks, this paper presents a detailed examination concerning the context, implications and possible derivative issues of these challenges.

## The challenges of transforming the TWB 3.0

Since 2012, the TWB has been evolving from the TWB 1.0 (quantity-oriented) to the TWB 2.0 (quality-oriented) [1]. Specifically, the TWB is no longer measured by the amount of biospecimens they collect, but by the utilization of these high-quality biospecimens to drive investigational research. As shown in Table 1, the TWB has promoted novel biomedical research. Approximately 150 studies derived from the TWB's resources have been published in important international journals with impact factors ranging from 0.97 to 28.349 (see Additional file 1). Furthermore, there have been many unanticipated benefits generated from the TWB’s research design. For instance, in the biobank’s original design, the TWB only recruited participants between 30 and 70 years old, with no prior history of cancer (currently, 130,093 cases have been received). However, follow-up tracking is carried out every 2–4 years, and cancer has been detected in more than 300 participants out of 29,432 follow-up cases. This data has provided important insights to the field of oncology/cancer research. In addition, the TWB has obtained International Organization for Standardization (ISO) 27001 and ISO 29100 certifications for governance and personal data protection, providing guarantees for biomedical research. Biobank 3.0 has been put forward as “a possible mode that may find a balance between the public welfare and business applications”. Obviously, this should be the main focus for the TWB 3.0 transformation going forward.

**Table 1:** Applications and publications using the TWB’s Resources from 2016 to 2020 June [25]

Year	Approved application	Publication	Publication/Application ratio	Range of impact factor
2020 (as of June 2020)	27	31	1.15	2.468–28.349
2019	24	68	2.83	0.97–8.689
2018	23	19	0.83	1.448–9.101
2017	20	23	1.15	2.170–14.079
2016	29	3	0.10	4.259–5.340
2015	16	2	0.13	2.133–14.921
2014	5	2	0.40	3.234–5.013

**Fig. 1:**
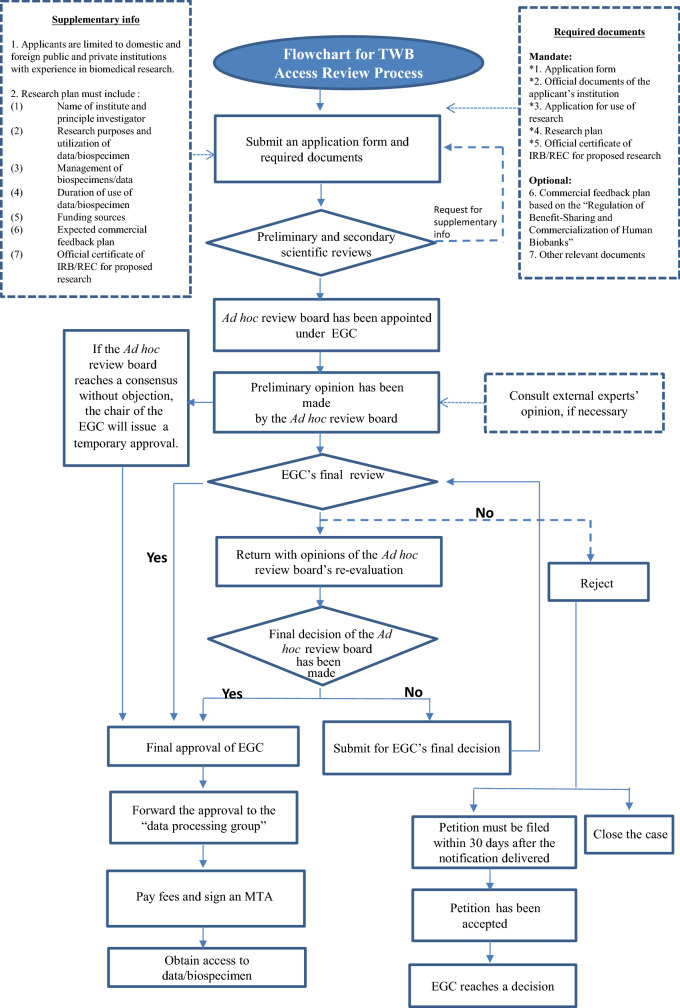
Application/review process of the TWB

As shown in Fig. 1, the current application process for accessing the TWB’s resources is very burdensome and time consuming. This is especially true for the Ethical Governance Committee (EGC) review process. While, in most of the cases, the TWB can grant the access to an application after the fast track ad hoc review process of EGC, a relatively complicated applicant’s case may need to wait until the final EGC decision. This can take a tremendous amount of the time, as the EGC only makes decisions on a quarterly basis. In addition, the TWB’s interaction and communication with participants is “limited” to the initial informed consent and subsequent regular follow-ups, making it difficult to make use of Big Data and/or AI applications. This also means that participants are prevented from developing an ongoing relationship with the TWB. Participant engagement is critical for the extensive application of the TWB’s resources in supporting cloud computing practices and/or precision medicine developments. Using All of Us [7] as an example, the TWB will take steps to not only improve its transparency but also revise its consent to either “e-consent” or “dynamic consent” to allow for participant’s active participation and encourage participants and biobank users to form long-lasting partnerships with the TWB.

Therefore, the TWB 3.0 may not only function as an integrated platform that balances public welfare with business innovation, but also meets the needs of external stakeholders and, further, allowing clinicians, researchers, and bioethicists to work together towards the goal of guaranteeing the right to use biospecimens [8]. However, to reconcile these needs with the overarching goal of the TWB 3.0 transformation, there are at least three challenges that need be overcome.

Now, let’s ask: “Is the TWB ready for the TWB 3.0?” The first challenge posed by the TWB 3.0 transformation is the difficulty bringing Taiwan’s 33 biobanks together for consolidation and/or collaboration purposes, or to be more specific, the issue of cross-database integration. Indeed, the Taiwanese government has already appointed the NHRI as the body responsible for integration, by charging them with the task of developing a virtual biobank platform similar to the Biobanking and BioMolecular Resources Research Infrastructure-European Research Infrastructure Consortium (BBMRI-ERIC) [9]. Taking the BBMRI-ERIC’s experience into consideration, its Ethical, Legal and Social Implications (ELSI) program includes “Quality & Management Mechanism” and “Navigator System” using the BBMRI-ERIC’s protocol for cross-database interface as a reference. Challenges associated with reconciling ELSI with the BBMRI-ERIC’s General Data Protection Regulation (GDPR) have drawn our attention to various data security issues as well, especially in terms of the regulation’s impact on cross border/databases sharing [10–13], data accessibility [14, 15] and data safety [11, 16, 17]. All these BBMRI-ERIC related cross-database integration issues have been part of the TWB 3.0 mandates, too.

That being said, this virtual biobank platform is meant to further solve the long-standing ELSI issues with linking the TWB’s genetic database with Taiwan’s nation-wide database of medical records created by the National Health Insurance Program [18]. In addition to the BBMRI-ERIC, the European Union experiences with GDPR compliances associated with obtaining and sharing personal data for research purposes [19] have also been taken into consideration by the NHRI. However, up until now, Taiwan’s cross-biobank interface protocol has not yet to create a harmonized standard for the collection, storage and sharing of biospecimens and data sets.

The second challenge associated with TWB 3.0 transformation relates to the effectiveness of the biobanking process and the efficiency of biospecimens/data release. The evolution of biobanks over time has shown that meeting the needs of various stakeholders and maintaining sustainable operations is at the core of biobank 3.0 [8]. A biobank should not only be big, but also needs to be efficient in order to deal with the whole biobanking process, including data/biospecimen collection, storage, release and access.

**Fig. 2:**
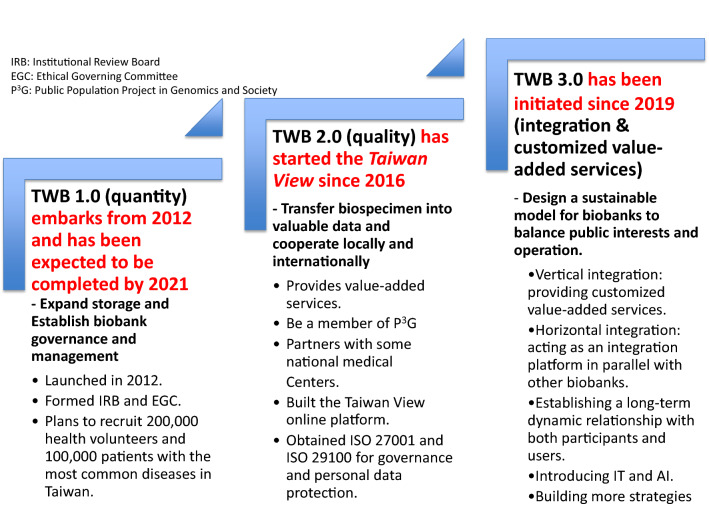
An illustration on the pathway from the TWB 1.0 to the TWB 3.0

It has been suggested by some scholars that there are three phases of biobank development [6]: the biobank 1.0 consists of establishing a biobank and finalizing its governance structures; the biobank 2.0 is associated with regional and/or international integration; the biobank 3.0 is characterized by efforts to achieve sustainability. Whereas the biobank 1.0 lays the foundation for expanding the number of data/biospecimens accrued and stored, the biobank 2.0 improves processing speed and ensures a higher quality of data/biospecimens. For these purposes, the cross data/border “ELSI compliance” and “scientific value” of data/biospecimens are enhanced. Now, all of the elements associated with the biobank 2.0 will be complemented by developing the TWB infrastructure; including the introduction of ISO certification, information security & efficiency enhancement, ELSI upgrade, integrated multi-center recruitment, the synthesis of data/information systems, international accessibility, etc. Figure 2 shows the pathway of moving the TWB from the biobank 1.0 to 2.0, and then to 3.0.

In terms of the TWB transformation, reducing application processing time might be a critical key to increasing the number of users and, accordingly, improving the utilities of the biobank. The UK Biobank [20], for instance, has cut its application time in half in 2020, from an average of 24 weeks to 12 weeks. This makes the UK Biobank a good example of the biobank 2.0 in terms of performance in regional and/or international accessibility. Indeed, this performance has engendered considerable social trust, making the UK Biobanks quickly launching a new COVID-19 research project, which aims to recruit 20,000 participants over the next six months (at least) [21]. Obviously, there is a considerable gap of processing speed between the TWB and the UK Biobank, which reflects a need for the TWB to improve its efficiency in processing applications. For example, over the past three years the UK Biobank has processed an average of 200 released applications per year. The Uppsala Biobank [22], a member of BBMRI-ERIC, processes about 100 applications per year. The TWB, on the other hand, only approved 24 applications in 2019 (as shown in Table 1). To improve its efficiency, the TWB has partnered with the National Center for High-performance Computing to establish a reliable data-releasing system. This system will integrate computing resources with personal data protection mechanisms and interface with various value-added services.

The third challenge associated with transforming the on-going TWB 2.0 to the TWB 3.0 relates to benefits, funding and sustainability. Operational efficiency, financial success and social trust are important key factors for evaluating the sustainability of biobanks [23]. As a biobank enters into the third phase, each biobank operator must weigh their own business advantages and risks in planning for the long-term survival. It is essential that biobanks are not just a combination of specimens, software and hardware. Rather, they must satisfy industrial needs and provide high value-added services to various stakeholders [4]. Just as in regular enterprise management, biobanks must seek sustainable choices to match their high investment costs and uncertainty of funding. In other words, the sustainable management of biobanks largely depends on whether these bodies can keep up with the times by providing new functions and roles, such as horizontal integration and vertical integration to the healthcare industry and/or engaging in regional or international harmonization of standards for the possibility of exchanging data globally [24]. The UK biobank is the one currently meeting this standard. Therefore, it is being used by the TWB as a benchmark, as outlined in the TWB 3.0 strategy mentioned below.

## The strategy and approach of the TWB 3.0

So far, many of the aforementioned challenges have not yet been solved. However, a biobank’s value can perhaps be best assessed by the contributions its data has made to the field of biomedical research. In the TWB’s case, although the TWB only approved 24 access applications in the year of 2019, as illustrated in Table 1, these applications resulted in 68 publications—many of which appearing in high-quality journals with high impact factors [25].

Furthermore, in light of a myriad of reports highlighted important social and economic values promoted by the UK biobank such as recently fighting against COVID-19 [26] and highly appraised by a 2018 feature in *Nature* magazine [27], we believe that the TWB 3.0 design should take into account how data drawn from biobanks can be practically transformed into valuable applications. The UK Biobank, All of Us and along with BBMRI-ERIC serving as good models of how the TWB 3.0 can operate in a more socially and financially responsible way. Therefore, this paper explores the sustainable approaches of these three biobanks, as these three organizations represent three main but different types of biobanks established around the world.

**Fig. 3:**
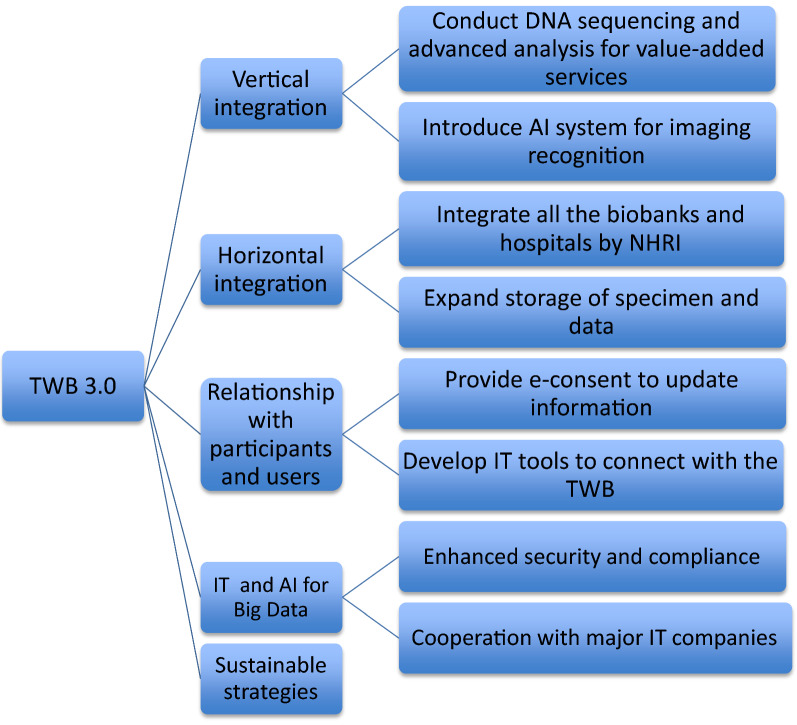
Strategy for transforming biospecimen/data into value-added service for achieving the TWB 3.0

After examining the UK Biobank, All of Us and BBMRI-ERIC’s respective relationships with the biomedical industry, we propose that the TWB should adopt both vertical and horizontal integration, as illustrated in Fig. 3. We also present some recommendations to bridge the gap between the TWB 2.0 and the TWB 3.0. These recommendations are part of a four-dimensional strategy to implement the TWB 3.0 transformation.

### Vertical integration: provide customized value-added services

The TWB should provide more customized value-added services to users to increase usage rate with heterogeneous integration. Furthermore, the efficiency of the application process for release needs to be improved so that it is in line with the biobank 3.0. Also, the TWB database’s speed of data transmission needs to keep up with global operations and large-scale development. As a result, the TWB has built an online platform called *Taiwan View* [28], an information platform carrying the summary data of whole genome sequencing and whole genome genotyping that incorporates full-fledged support for novel-generation biobanking, including chromosome, position, single nucleotide polymorphism (SNP) ID, reference allele, alternative allele, genotype counts and call rate. *Taiwan View* can provide an online query webpage for SNP information and an online genome-wide association study (GWAS) that allows users to upload their genotype data and perform GWAS using the Cochran–Armitage trend test. So far, the value-added services available for public access on *Taiwan View* can be found in Table 2 [29].

**Table 2:** Information of value-added services in the Taiwan View [29]

Value-added services	Number of available data	Platform
Genomics		
Whole-genome genotyping	TWB Chip 1.0: 28,690 TWB Chip 2.0: 83,220	TWB Chip 1.0: Axiom Genome-Wide Array Plate with 653,000 SNPs TWB Chip 2.0: Axiom Genome-Wide Array Plate with 750,000 SNPs
Whole-genome sequencing	2020	Thermo Fisher Ion Proton & Illumina
Human leukocyte antigen (HLA) typing	1108	NXType NGS Reagents Class I: HLA-A, HLA-B, HLA-C, Class II: HLA-DPA1, HLA-DPB1, HLA-DQA1, HLA-DQB1, HLA-DRB1, HLA-DRB345
Epigenomic		
DNA methylation	2112	Illumina Infinium MethylationEPIC BeadChip
Metabolomics		
Human blood metabolome	768	Bruker Avance 800 Nuclear Magnetic Resonance (NMR) spectroscopy

In recent years, Big Data has played an increasingly important role in the development of precision medicine. However, one of the challenges facing biobanks in public healthcare is how to improve biomedical/genetic research and the predictive analytics of Big Data with information technology (IT) and artificial intelligence (AI) [30–32]. IT, Taiwan’s main core competence, now mainly focuses on AI applications. Therefore, through the help of IT and AI, Big Data of the integrated platform of biobanks can gradually grow to meet each user’s needs. They possess the ability to customize research tools, which can increase user incentive. In particular, medical imaging tools, such as X-ray photography, computer tomography, and magnetic resonance imaging (MRI), have lower background noise than other imaging data. They are more suitable for the application of AI image recognition. Therefore, many governments, such as the United Kingdom, Canada, Japan, and India, have promoted national AI policies for medical imaging to assist physicians in diagnosis. For instance, in 2018, the US Food and Drug Administration (FDA) approved an AI medical imaging device to detect certain diabetes-related eye problems [33]. Recently, the Wellcome Trust found a research developing an AI-aided method to detect glaucoma progression using Detection of Apoptosing Retinal Cells (DARC) 18 months earlier than the current gold standard method [34].

Let’s take the UK Biobank as an example. The UK Biobank has released their resources of biospecimens since 2012 and completed a follow-up survey of 20,000 participants in 2013, including a series of health data with participants and regular receipts of relevant health information (e.g., death, cancer and medical treatment). In addition, the UK Biobank has committed to the value-added services of its resources, including: (1) biomarkers for providing more rapid and high-value research tools for biomedical research, (2) exome sequencing for 50,000 participants in cooperation with GlaxoSmithKline, (3) an MRI study that expects to collect images of 100,000 participants’ internal organs, (4) a physical monitor whereby 100,000 participants are asked to wear a monitor device that provides biological information for all activities undertaken during a 24-hour period, and (5) a heart monitor pilot study that monitors participants over the age of 65 and measures their heart activity, heart beats, and other related issues experienced within a two week period through the Zio patch [35].

Recently, the UK Biobank has also upgraded its IT system to optimize its database content and usability. The UK Biobank’s data center is located at Oxford University. Combined with Big Data, it allows more researchers to use UK Biobank resources. Currently, there are 7500 registered researchers in UK Biobank. These researchers can further analyze, optimize and enhance the service efficiency of the UK Biobank to ensure more accurate and comprehensive research. Furthermore, the UK Biobank offers an innovative platform to attract international companies, such as GSK, Regeneron, AbbVie, Alnylam Pharmaceuticals, AstraZeneca, Biogen, Pfizer, Takeda and Bristol-Myers Squibb, to provide research materials. Up until 2020, there are more then 750 published papers that have drawn upon the UK Biobank’s resources. The UK Biobank has also recently adjusted its organizational structure. First, they introduced the Ethics Advisory Committee (EAC) to advise the board on new ethical issues relating to governance, research, and the implementation of IT or other novel technologies for health [36]. This allows the UK Biobank to keep pace with increasingly large and complex database operations, and ensure the efficient use of its resources.

The application of AI medical image recognition has also started to gradually emerge in Taiwan. In addition to conducting breast ultrasounds, DeepMets, an imaging technology invented by the Taipei Veterans General Hospital, can also examine brain tumors through an AI interpretation system [37]. Another AI product—DEEP01 proposed by the Einstein Artificial Intelligence Corporation also has the capability to diagnose cerebral hemorrhages via AI, ensuring the rapid and accurate interpretation of the computer tomography of patients [38]. Since 2017, the Ministry of Science and Technology of Taiwan has begun to construct medical imaging cases (including cardiac tomography, brain metastases, acoustic neuroma, lung cancer, computer tomography, blood vessels, etc.) in collaboration with National Taiwan University, Taipei Medical University, and the Taipei Veterans General Hospital. A national grid center will soon be established for AI medical research, along with its development and application. However, at present, data sharing has so far been limited to these three institutes. To this end, the TWB could collect the relevant medical imaging data of existing participants’ X-ray photography, computed tomography, MRI, etc. By adopting the UK Biobank’s model, the TWB could accelerate the development of medical AI in Taiwan through integrating medical image data with existing genetic data.

### Horizontal integration: in parallel with other biobanks, acting as a part of the NHRI integration platform

The Human Biobank Management Act of Taiwan was passed in 2010, and provides legal regulations for the collection, processing and use of biospecimens in Taiwan [39]. Currently, 33 biobanks have been approved and established. However, the scales and the standards of these institutions are different, and so far there has been no harmonization. As a result, integrating data from these 33 biobanks, ~ 460,000 participants, ~ 4.5 million biospecimens, and major hospitals is critically important. Through the establishment of the national-level platform, various databases (e.g., the National Health Insurance’s electronic medical records, cancer registration, rare diseases, and other local biobanks’ databases) can be effectively integrated and connected, and the application of precision medicine can be accelerated.

NHRI is in charge of integrating 33 Taiwanese biobanks with this standardization resulting in the formation of a large infrastructure network, similar to BBMRI-ERIC. Here, let’s take BBMRI-ERIC as an example. BBMRI-ERIC consists of 19 European Member States and 1 international organization—the International Agency for Research on Cancer. This means that BBMRI-ERIC takes a top-down governance strategy involving a central node to integrate the Pan-European Research Infrastructure for health research [40]. So far, the BBMRI-ERIC directory integrates 100 million biospecimens and provides a roadmap of their partner biobanks. A central office supervises this kind of network biobank. Each partner biobank stores biospecimens under a series of standardized and approved conditions. The privacy of each biospecimen is protected, and the data can be shared and repeated by partner biobanks.

The goals of integrating this national-level platform to provide support for multi-site, multi-investigator collaborative studies are as follows: (1) creating an integrated platform for each biobank, (2) establishing a single application window and review process so that interested applicants can apply for biometric data through this platform, (3) providing value-added services for data/biospecimens, and (4) offering appropriate rewards for biobanks and biobank users to encourage them to return research data to the integrated platform to increase the content of the human biological database. More specifically, the genetic and health data from the partner biobanks will be gathered by the NHRI’s central office’s IT system to make the best use of this research. Also, 50% of feedback funding will be re-distributed to the partner biobanks. In addition, the central office will play a multifunctional integration role to integrate international or domestic biomedical research. Moreover, all the data collection, operation specifications, review processes and utilization should be under the same ELSI, code of conduct, and Standard Operation Procedure (SOP).

Therefore, with this strategy of horizontal integration, the TWB (as one of the partner biobanks) will benefit from improving its release usage, because the value-added services proposed by the TWB linked with this horizontal integration platform will attract more users to apply through any partner biobank for the release of data/biospecimens from the TWB. Furthermore, the TWB’s database can be further transformed into a “know-how” service-oriented company. The UK Biobank provides a good example through its establishment of the UK Biocentre Ltd., which allows the Biobank to extend its expertise in managing biological databases/biobanks to other domestic biological database research projects [41].

In brief, this national-level horizontal integrated platform will be an important source of information for biomedical research and precision medicine. It will also accelerate disease research and new drug development, and attract international pharmaceutical companies to invest and improve the quality and standard of medical care in Taiwan.

### Establishing a long-term dynamic relationship with both participants and biobank users

All of Us utilizes a highly interactive biobank model that treats participants as partners who participate directly in database operation and enrich research orientation and capacity. First of all, the Participant Center of All of Us manages the enrollment of direct volunteers, so that people who want to join the All of Us biobank do not have to have access to a participating health care provider organization. In addition, All of Us provides some IT tools and platforms to help participants “enroll” in the program, “share” their health information, and “receive” updates. Finally, the All of Us genome center generates genomic data from the biospecimens of participants. Some of the centers will also analyze this data for genetic results and return them to participants.

To compare, All of Us and the UK Biobank both have dynamic interaction with participants and users, whereas the BBRMI-ERIC and the TWB do not. All of Us has dynamic, instant, and long-term interaction with participants. For example, All of Us reports feedback results to participants, including the return of genetic information. All of Us also customizes service for users. Furthermore, the UK Biobank has convenient search tools, quick access to information, the rich value-added services of specimens and data, and digital platforms for users. In addition, the UK Biobank has long-term interaction with participants (e.g., informed consent/re-contact) and, under its regulations, reports limited incidental findings to participants.

BBMRI-ERIC, on the other hand, provides users with a convenient method of data retrieval through their well-developed directory. Also, BBMRI-ERIC has indirect interaction with participants, who must be contacted through each biobank independently. It should be noted that BBMRI-ERIC has developed the colorectal cancer cohort to collect over 10,000 colorectal cancer datasets from across Europe. Still, a virtual biobank like BBMRI-ERIC has been limited in terms of its interactions with participants recruited from various member biobanks. In turn, it has to count on real biobanks’ cooperation and/or collaboration in contacting each participant, in order to accumulate more relevant data and further enhance their ability to customize services for “valuable users”, e.g., leading pharmaceutical companies.

Indeed, it is convincible that the promotion of the “valuable users” is one of the most important elements of biobank sustainability. As we have seen from the UK’s experiences, a good data quality management system that allows for the expansive application of advanced technology and research, e.g., MRI brain scans for the gene and neurological disease association, is an essential aspect of achieving this goal [42]. For instance, as a result of these enhanced value-added data services, the UK Biobank is able to attract funding from the top worldwide leading pharmaceutical companies, which pay to access their data [43].

As we found in a previous investigation [18], while the management of “incidental findings” did generate a new set of ELSI issues for biobank operations, the depth (category) and breadth (scale) of biobanks’ high value-added services are supported and trusted by participants and citizens. This seems to be the result of “altruism”—especially in large cohort studies. Learning from All of Us and the UK biobank, the TWB’s interactions with participants and users should be more expansive and extensive than its traditional methods of passive consent and periodic follow-up. Dynamic consent models help to establish interaction among participants. Thus, the TWB might consider upgrading its informed consent process to benefit participants during routine tracking, cooperating with the new database management system, and introducing e-consent. The TWB might also seek to strengthen partnerships with participants. In addition to fostering deeper trust and support, this should also be conducive to the promotion and implementation of information value in the future. Therefore, implementation strategies should be designed to keep participants and biobank users engaged over the longer term.

### Introducing IT and AI for Big Data in precision medicine

If the TWB wants to introduce a strategy of vertical and horizontal integration, then it needs to upgrade its management system and accumulate more data in order to realize the benefits of big data. For example, through AI, the TWB can optimize its management of biospecimens, data storage and quality. The TWB can consider adopting the following two measures.

#### 1. Enhanced security and compliance by introducing cloud computing services

The UK Biobank sets a good example for the TWB. Before introducing cloud computing, the UK Biobank explicitly established the Cloud Computing Policy [44]. We believe that portions of this policy are significantly relevant to the TWB, especially the sections regarding data authorization and protection. The UK Biobank’s Cloud Computing Policy requires that cloud-computing providers comply with the UK Biobank’s Material Transfer Agreement (MTA) and other regulations that pertain to the release and use of data. We agree that ensuring compliance with a patient’s informed consent and regulatory guidelines should be the TWB’s first priority. Furthermore, their policy also expects that cloud service providers uphold information security industry standards (e.g., ISO 27001, ISO 27017 and ISO 27018), and the data is protected with encryption management while it is both at rest and in transit (Advanced Encryption Standard (AES) 256 recommended). We acknowledge that confidentiality is of paramount concern for biobanks, as a security breach or personal data leak could have an adverse impact on public trust. One means of alleviating these concerns would be for the TWB 3.0 to utilize new IT tools, such as blockchain, allowing for the secure and ethical transfer of biospecimens to researchers [45]. In brief, we suggest that the TWB should first set up autonomy regulations and establish solid protection measures for participants before introducing cloud computing services.

#### 2. Cooperation with major IT companies

The role of IT in biobanking not only involves using AI to access and analyze the big data of biospecimens [46]. IT can also ensure that the privacy of individual health information is maintained and that the security of electronic systems is preserved [47]. Although the regulations of releasing biospecimens/data do not prevent commercial companies with research capabilities from filing an application, so far only a few companies engaged in biomedical research have applied data from the TWB. Today, with the recent demand for the use of big data in medical research, more and more IT companies are involved in biological databases (e.g., Google, Microsoft, Samsung used to apply data from the UK Biobank). Therefore, it is worth considering how the TWB can more effectively attract the IT industry.

### Introducing sustainable financial strategies

In order to introduce IT and AI into the TWB’s operations, the maintenance and management costs of the corresponding machines and equipment should be taken into consideration. However, at the same time, IT and AI related companies have the financial means to support the TWB 3.0 transformation. In order to find “a balance between public welfare and business operations”, as mentioned above, Taiwan’s ‘‘Regulation of Benefit-Sharing and Commercialization of Human Biobanks’’ should be revised so that it encompasses an extra fee or tariff charge for customized services provided by IT and AI companies in exchange for biobank database usage [1]. While this approach might help ensure financial sustainability of the TWB 3.0 transformation, as most biobanks around the world know, there are still many issues associated with ensuring a fully sustainable future.

Most biobanks are sponsored by charity organizations and/or governments. Their funding situations can be identified on the web; for instance; the UK Biobank is funded primarily by the Wellcome Trust and the Medical Research Council (MRC). Both organizations have provided the biobank with funds to plan, roll out and maintain their research program, and to top-up their resources as their research has matured [48]. Similarly, All of Us is sponsored by the US National Institutes of Health in partnership with the Mayo Clinic in Rochester, Minnesota. In addition to creating a specialized facility for the All of Us Research Program, the Mayo clinic also supports the collection, analysis, storage, and distribution of the biosamples from the All of Us research program [49]. In Taiwan, only very few biobanks are claimed to be privately owned; most biobanks are fully or partially funded by the government. As previously reported, we also provided additional financial strategies to address sustainability issues in biobanking using the TWB as an example [1].

## Conclusion

Similarly to many of the leading biobanks in the world, the TWB has come a long way over the past eight years. The institution has moved away from its initial “establishment” and the goal of fostering “regional and international collaboration”, and then is now striving for a “sustainable paradigm”. In order to achieve the sustainable future, several measures should be adopted to facilitate the TWB’s transformation from the TWB 2.0 to the TWB 3.0. When assessing a biobank’s evolution, it is worth pointing out that biobanking practices have shifted from statistical data collection and storage to an AI driven platform, and also from a user-centered operation to a person-centered approach with user-friendly management. Therefore, we believe that some of the innovative measures undertaken by BBMRI-ERC, the UK Biobank and All of Us are the best strategy for the TWB 3.0 transformation.

**Fig. 4:**
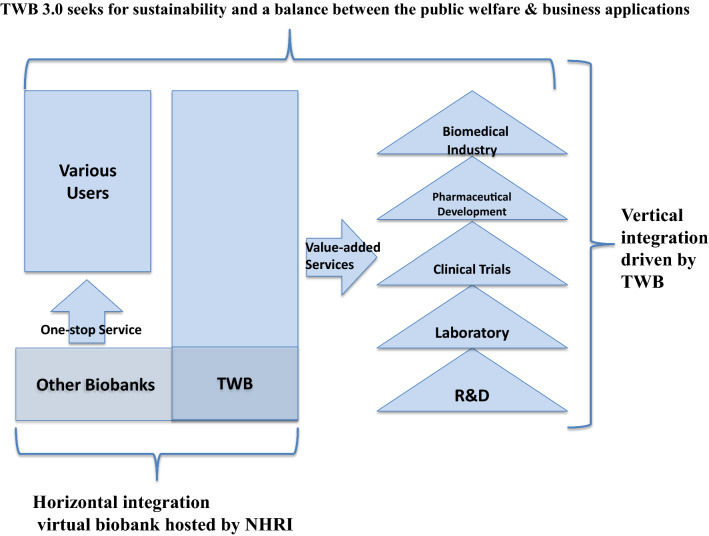
A schematic diagram to demonstrate our proposed vertical and horizontal integration

Firstly, as shown in Fig. 4, the TWB 3.0 (phase 3) cannot be accomplished without the required vertical and horizontal integration among various biobanks—which has been the main goal of the TWB 2.0 (phase 2). Learning from the BBMRI-ERC, the NHRI of Taiwan will be in charge of developing a horizontal collaboration network of Taiwan’s 33 biobanks through the platform of Taiwan’s Virtual Biobank. To complement the goal of TWB 3.0, the TWB must join this horizontal platform, and, by taking the UK Biobank and All of Us’ designs into account, match it with the required vertical integration. This should be done through efforts to bridge the gap between participants and users through service quality vis-à-vis recruitment quantity improvement, as exemplified by the BBMRI-ERC. The ambitious design of All of Us, which presents a comprehensive solution for all stakeholders, should be considered as a long-term goal of TWB 3.0 as well.

Secondly, various ELSI concerns need be addressed at the TWB 3.0 phase, so as to meet the needs of Big Data Era and precision medicine-related research. For instance, in line with the practices of the UK Biobank and All of Us, measures to increase the number of participants should be undertaken through developing an enhanced and dynamic consent mode to encourage participants’ feedback and/or active contributions on a continuing basis. This will further promote the partnership relationship between the TWB and its participants, which is critical for the development of precision medicine and Big Data Era research. Big Data raises new challenges in terms of privacy infringement and information security concerns. It therefore will be necessary for the TWB 3.0 to take enhanced and innovative information security protocols and privacy protection measures into consideration, especially when upgrading its IT infrastructure and Ethical Governance Framework.

Thirdly, the TWB’s finance structure needs significant improvement. The TWB 3.0 should take the lead of the UK Biobank and All of Us and adopt a public–private funding structure. In addition to a stable public funding support, the TWB 3.0 should secure its stable public funding support by upgrading its data value and working out a reasonable reimbursement protocol through ethically acceptable industrial applications and/or collaboration mechanisms with users. This will be one of the most challenging issues for the TWB in the years ahead.

## Supplementary information

**Additional file 1.** Impact factors of published journals derived from the TWB’s resources

## Data Availability

Not applicable.

## Abbreviations

AES: advanced encryption standard
AI: artificial intelligence
BBMRI-ERIC: Biobanking and BioMolecular Resources Research Infrastructure-European Research Infrastructure Consortium
CAGR: compound annual growth rate
DARC: detection of apoptosing retinal cells
EAC: Ethics Advisory Committee
EGC: Ethics and Governance Council
ELSI: Ethical, Legal, and Social Implication
FDA: Food and Drug Administration
GDPR: General Data Protection Regulation
GWAS: Genome-Wide Association Study
HLA: Human Leukocyte Antigen
ISO: International Organization for Standardization
IT: Information Technology
MRC: Medical Research Council
MRI: Magnetic Resonance Imaging
MTA: Material Transfer Agreement
NIH: National Institutes of Health
NHRI: National Health Research Institutes
SNP: Single Nucleotide Polymorphism
TWB: Taiwan Biobank
